# Steps to ensure accuracy in genotype and SNP calling from Illumina sequencing data

**DOI:** 10.1186/1471-2164-13-S8-S8

**Published:** 2012-12-17

**Authors:** Qi Liu, Yan Guo, Jiang Li, Jirong Long, Bing Zhang, Yu Shyr

**Affiliations:** 1 Center for Quantitative Sciences, Vanderbilt University School of Medicine, Nashville, TN 37232, USA; 2 Department of Biomedical Informatics, Vanderbilt University School of Medicine, Nashville, TN 37232, USA; 3 Vanderbilt Epidemiology Center, Vanderbilt University, Nashville, TN 37232, USA; 4 Department of Cancer Biology, Vanderbilt University School of Medicine, Nashville, TN 37232, USA; 5 Department of Biostatistics, Vanderbilt University School of Medicine, Nashville, TN 37232, USA

## Abstract

**Background:**

Accurate calling of SNPs and genotypes from next-generation sequencing data is an essential prerequisite for most human genetics studies. A number of computational steps are required or recommended when translating the raw sequencing data into the final calls. However, whether each step does contribute to the performance of variant calling and how it affects the accuracy still remain unclear, making it difficult to select and arrange appropriate steps to derive high quality variants from different sequencing data. In this study, we made a systematic assessment of the relative contribution of each step to the accuracy of variant calling from Illumina DNA sequencing data.

**Results:**

We found that the read preprocessing step did not improve the accuracy of variant calling, contrary to the general expectation. Although trimming off low-quality tails helped align more reads, it introduced lots of false positives. The ability of markup duplication, local realignment and recalibration, to help eliminate false positive variants depended on the sequencing depth. Rearranging these steps did not affect the results. The relative performance of three popular multi-sample SNP callers, SAMtools, GATK, and GlfMultiples, also varied with the sequencing depth.

**Conclusions:**

Our findings clarify the necessity and effectiveness of computational steps for improving the accuracy of SNP and genotype calls from Illumina sequencing data and can serve as a general guideline for choosing SNP calling strategies for data with different coverage.

## Background

Next-generation sequencing (NGS) technology is a powerful and cost-effective approach for large-scale DNA sequencing [1]. It has significantly propelled the sequence-based genetics and genomics research and its downstream applications which include, but are not limited to, de novo sequencing [2,3], quantifying expression level s[4-7], providing a genome-scale look at transcription-factor binding [8,9], creating a foundation for understanding human disease [10-12] and systematically investigating of human variation [13,14]. A number of projects based on NGS technology are underway. For example, 1000 Genomes Project http://www.1000genomes.org/ aims to provide a comprehensive resource of human genetic variation as a foundation for understanding the relationship between genotype and phenotype [14]. The NHLBI GO Exome Sequencing Project (ESP) http://evs.gs.washington.edu/EVS/ focuses on protein coding regions to discover novel genes and mechanisms contributing to heart, lung and blood disorders. TCGA (The Cancer Genome Atlas) http://cancergenome.nih.gov/ has been sequencing a large number of tumor/normal pairs to provide insights into the landscape of somatic mutations and the great genetic heterogeneity that defines the unique signature of individual tumor [15]. The ability to discover a comprehensive list of human genetic variation and to search for causing variation or mutation underlying diseases depends crucially on the accurate calling of SNPs and genotypes [16].

Translating the raw sequencing data into the final SNP and genotype calls requires two essential steps: read mapping and SNP/genotype inference. First, reads are aligned onto an available reference genome, then variable sites are identified and genotypes at those sites are determined. SNP and genotype calling suffers from high error rates that are due to the following factors. Poor quality or low-quality tails prevent reads from being properly mapped. Each read is aligned independently, causing many reads that span indels are misaligned [17]. The raw base-calling quality scores often co-vary with features like sequence technology, machine cycle and sequence context and, thus, cannot reflect the true base-calling error rates [17]. These alignment and base-calling errors propagate into SNP and genotype inference and lead to false variant detection. Moreover, low-coverage sequencing always introduces considerable uncertainty into the results and makes accurate SNP and genotype calling difficult. To obtain high quality SNP and genotype data, most contemporary algorithms use a probabilistic framework to quantify the uncertainty and to model errors introduced in alignment and base calling [17-20]. In addition, a number of optional steps are recommended. Some are prior to variant calling, including raw reads preprocessing, duplicate marking, local realignment, and base quality score recalibration[17]. Others are posterior to variant calling, including linkage-based genotype refining [21-23] and SNP filtering [24] or variant quality score recalibration [17].

Here we focused on those optional steps preceding variant calling. We assessed their relative contributions and evaluated the effect of their orders on the accuracy of SNP and genotype calling with data generated on Illumina sequencing platform, which is currently the most widely used sequencing technology. Besides, we also compared the performance of three popular multi-sample SNP callers, SAMtools [20], GATK [17], and GlfMultiples [14], in terms of dbSNP rate, transition to transversion ratio (Ti/Tv ratio), and concordance rate with SNP arrays (Methods section). Our findings can serve as a general guide for choosing appropriate steps for SNP and genotype calling from Illumina sequencing data with different coverage.

## Methods

### Sequencing data and SNP calling

Five samples were selected for whole exome sequencing. All samples were taken from women with very early-onset (22-32 years old) breast cancer or early-onset (38-41 years old) plus a first-degree family history of breast cancer [25].

Genomic DNA from buffy coat was extracted using QIAmp DNA kit (Qiagen, Valencia, CA) following the manufacture's protocol. Exonic regions were captured using Illumina TruSeq Exome Enrichment Kit. It targeted 201,071 regions (62.1 million bases; 49.3% inside exons; average length 309 bp), covering 96.5% of consensus coding sequence database (CCDS). An Illumina HiSeq 2000 was used to generate 100-bp paired-end reads (five samples per lane).

Reads were mapped to the NCBI Build 37 reference genome with BWA [26], sorted and indexed with SAMtools [20]. Those reads were classified into three categories by their mapped locations on the genome, inside target regions, outside target regions with ≤ 200 bp distance and outside target regions > 200 bp distance. For these five samples, there was an average of 43.4% bases (42.7-43.7%) mapped to target regions, 21.4% (21.3-21.7%) mapped to outside ≤ 200 bp regions, and 35.2% (34.6-36.2%) mapped to outside > 200 bp regions(Table 1). As expected, the depth of coverage was the highest for inside target regions (~60× coverage per sample on average) and lowest for outside > 200 bp regions (~4× coverage per sample on average) (Table 1). 98.8% target regions, 92.1% of outside ≤ 200 bp regions and 58.3% of outside > 200 bp regions are accessed by sequencing data (Table 1).

**Table 1:** Summary of bases distribution for five samples whole-exome sequencing data

Coverage	Sample	Total mapped bases (Gb) (%)	Mean mapped depth (×)	Bases accessed(Gb) (% of genome regions)
High (Inside target)	1	3.71 (43.7%)	60.53	0.61(98.8%)
	2	3.75 (43.7%)	61.11	
	3	3.88 (43.5%)	63.27	
	4	3.90 (42.7%)	63.57	
	5	3.85 (43.4%)	62.71	

Medium (outside≤200 bp)	1	1.84 (21.7%)	30.05	0.74(92.1%)
	2	1.85 (21.5%)	30.15	
	3	1.91 (21.4%)	31.14	
	4	1.93 (21.1%)	31.40	
	5	1.89 (21.3%)	30.82	

Low (outside > 200 bp)	1	2.94 (34.6%)	3.99	1.66 (58.3%)
	2	2.99 (34.8%)	4.03	
	3	3.12 (35.1%)	4.18	
	4	3.30 (36.2%)	4.31	
	5	3.13 (35.3%)	4.16	

Poor-quality tails of reads were dynamically trimmed off by the BWA parameter (-q 15). Duplicated reads were marked by Picard. Base quality recalibration and local realignment were carried out using Genome Analysis Toolkit (GATK) [17,27]. SNPs were called simultaneously on five samples by GATK Unified Genotyper, SAMtools Mpileup and GlfMultiples using bases with base quality≥20 and reads with mapping quality ≥20.

### Definition of performance metrics

#### dbSNP rate

The percentage of variants found in dbSNP database [28](dbSNP rate) is used to measure an approximate false-positive rate of SNP calling. Here dbSNP 129 was used, which contains approximately 11 million SNP entries [29-31]. It excludes the impact of the 1000 Genomes project and is useful for evaluation. Multi-sample SNP calling is able to find more rare variants than single sample calling, thus the aggregate dbSNP rate is lower. Of ~640 k variants discovered from these five samples, about 77% were already catalogued in dbSNP 129 (Table 2). It should be noted that dbSNP rate is not an absolute measurement of which variant calls are better, but the same number of variants with higher dbSNP rate may reasonably suggest lower false-positive rates.

**Table 2:** Effects of data preprocessing on SNP calling accuracy

Call set (QUAL > = 50)	Site discovery					
	
	No. SNPs				Ti/Tv ratio	
	
	All	Known	Novel	dbSNP%	Known	Novel
raw	640946	499377	141569	**77.91%**	2.19	**1.65**
filterY	630641	490722	139919	77.81%	2.19	**1.65**
trim	651391	502951	148440	77.21%	2.18	1.58
filterY&trim	640487	493741	146746	77.08%	2.18	1.58

raw: without any preprocessing steps; filterY: removing those reads that fail the Illumina chastity filter; trim: trimming off low-quality tails from reads with the BWA parameter (-q 15); filterY&trim: removing those reads that fail the Illumina chastity filter and trimming off low quality tails. SNPs were called for five samples together by GATK using bases with base quality≥20 and reads with mapping quality ≥20. Only sites with QUAL > = 50 were considered as potentially variable sites.

#### Transition/transversion ratio

The variants are observed either as transitions (between purines, or between pyrimidines) or transversions (between purines and pyrimidines). The ratio of the number of transitions to the number of transversions is particularly helpful for assessing the quality of SNP calls [17]. Ti/Tv ratios are often calculated for known and novel SNPs separately. The expected Ti/Tv ratios in whole-genome sequencing are 2.10 and 2.07 for known and novel variants, respectively, and in the exome target regions are 3.5 and 3.0, respectively [17]. The higher Ti/Tv ratio generally indicates higher accuracy. When detected variants demonstrate a ratio closer to the expected ratio for random substitutions (e.g. ~0.5), low-quality variant calling or data is implied.

#### Genotype concordance

All five samples have been genotyped using the Affymetrix SNP 6.0 array in a previous genome-wide association study [25]. Detailed genotyping methods and stringent quality control criteria were described in Zheng et al., [25]. The original scan included three quality control samples in each 96-well plate, and the SNP calls showed a very high concordance rate (mean 99.9%; median 100%) for the quality control samples.

Genotypes obtained from the sequencing data were compared with those from the SNP array. The non-reference discrepancy (NRD) rate was used to measure the accuracy of genotype calls, which reported the percent of discordant genotype calls at commonly called on-reference sites on the SNP array and exome-sequencing. The mathematical definition of NRD can be found in Depristo et al., [17]. The lower NRD generally indicates higher accuracy of genotype calls.

## Results

### Effects of data preprocessing

**Figure 1:**
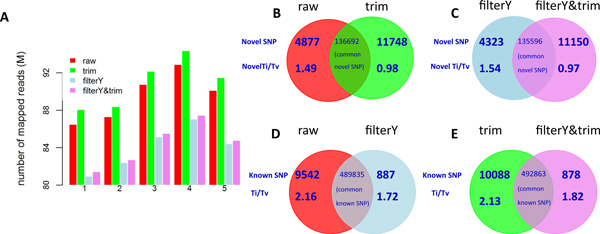
**Effects of read preprocessing steps on SNP calling**. Number of mapped reads using different preprocessing steps for five samples (A). Venn diagrams comparing identified novel variants between the raw call set and the call set with the trim step. Number of unique novel SNPs, the Ti/Tv ratio and number of common novel SNPs were listed(B). Venn diagrams comparing identified novel variants between the call set with the filterY step and the call set with both filterY and trim steps. Number of unique novel SNPs, the Ti/Tv ratio and number of common novel SNPs were listed (C). Venn diagrams comparing identified known variants between the raw call set and the call set with the filterY step. Number of unique known SNPs, the Ti/Tv ratio and number of common known SNPs were listed (D). Venn diagrams comparing identified known variants between the call set with the trim step and the call set with both filterY and trim steps. Number of unique known SNPs, the Ti/Tv ratio and number of common known SNPs were listed (E).

Using high-quality reads is expected to identify true variants. Generally, there are two ways to extract high-quality reads from Illumina sequencing data: removing reads that fail the Illumina chastity filter (filterY) and trimming off low-quality ends from reads (trim). The trim step obtained the largest number of mapped reads, while the filterY produced the fewest number of mapped reads resulting from lots of low-quality reads being discarded (Figure 1A). Although the trim step helped align more reads and identify slightly more variants (1.6%, ~651 k vs. ~641 k), it obtained a lower dbSNP rate (77.21%) and a lower novel transition/transversion ratio (Ti/Tv ratio) (1.58) compared with those using raw sequencing data (dbSNP: 77.91%, novel Ti/Tv ratio: 1.65) (Table 2). Trimming low-quality tails added 11,748 novel variants, representing about 8% of all novel calls, with a Ti/Tv ratio of 0.98, while it eliminated 4,877 novel variants with a Ti/Tv ratio of 1.49 from the raw call set (Figure 1B). The novel variants unique to the trim call set had a much lower Ti/Tv ratio (0.98) compared with the Ti/Tv ratio (1.49) of those unique to the raw call set, which suggested that more false positive variants were introduced by the trim step. Results from applying both filterY and trim steps (filterY&trim) compared with those from performing filterY step alone also revealed that trim step would increase the number of false positives (Table 2 and Figure 1C).

The filterY step identified fewer variants (~630 k); however, those variants showed the similar dbSNP rate (~77.8%) and Ti/Tv ratio (2.19 and 1.65, respectively) compared with the raw call set. Removing poor-quality reads from raw data (filterY) added 887 known variants with a Ti/Tv ratio of 1.72, while it eliminated 9542 known variants with a Ti/Tv ratio of 2.16 from the raw call set (Figure 1D). That is, filterY step dropped more than 8,000 known variants, representing about 2% of all known calls. These results suggested that throwing out those poor quality reads which failed the chastity filter might not be necessary for further SNP calling. Comparison results from applying both filterY and trim steps (filterY&trim) with those from performing trim step alone also revealed the useless of filterY step on improving SNP calling performance (Table 2 and Figure 1E).

A comprehensive comparison using variable quality thresholds for high-coverage data (inside target regions, ~60× coverage per sample on average, Table 1), medium-coverage data (outside regions with ≤ 200 bp distance, ~30× coverage per sample on average, Table 1) and low-coverage data (outside regions with > 200 bp distance, ~4× coverage per sample on average, Table 1) came to the same conclusion, that these two preprocessing step, filterY and trim, could not improve the performance of SNP calling, a conclusion contrary to the usual expectation. Application of the trim step might even introduce false positives, especially for high-coverage data. Compared with low coverage data, the problem of introducing false positives caused by the trim step is more serious for high coverage data (Additional file 1).

### Effects of duplicate marking, realignment and recalibration

**Figure 2:**
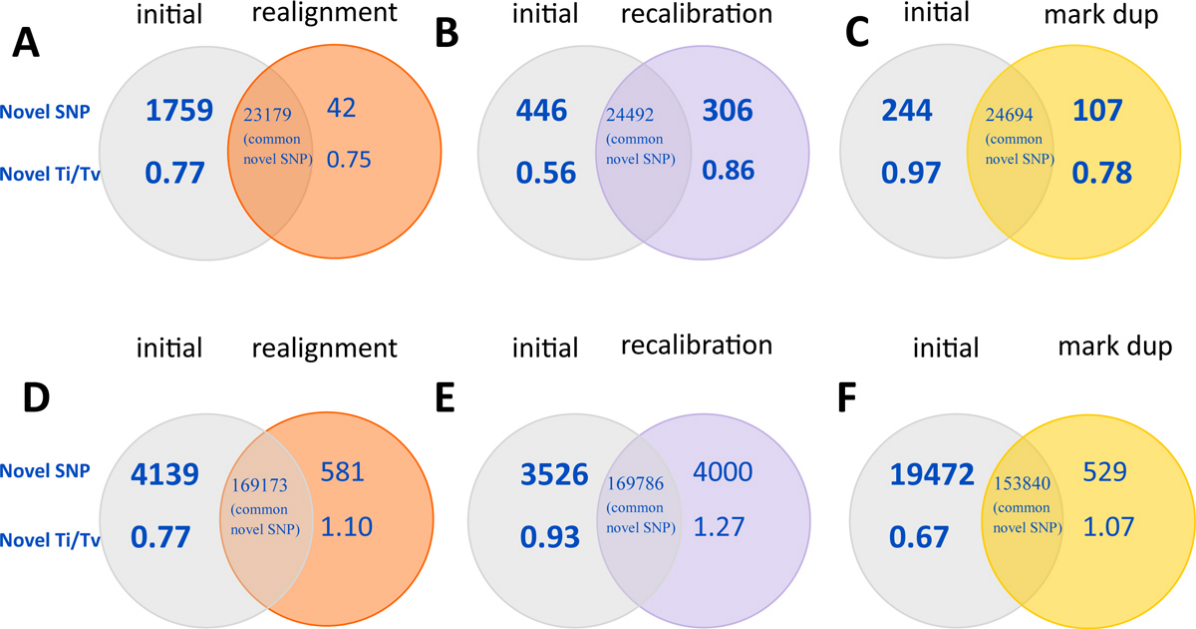
**Effects of realignment, recalibration and marking duplication on SNP calling from high and low coverage data**. Venn diagrams comparing identified novel variants between two call sets using different steps. Number of novel SNPs, the Ti/Tv ratio and number of common novel SNPs were listed in the comparisons between the initial call set and the call set with realignment step for high (A) and low (D) coverage data, between the initial call set and the call set with recalibration step for high (B) and low (E) coverage data, between the initial call set and the call set with marking duplication step for high (C) and low (F) coverage data.

Among the three optional steps, local realignment, marking duplication and base quality recalibration, local realignment obtained the highest dbSNP rate (75.45%) and novel Ti/Tv ratio (1.84) for high-coverage data (inside target regions, ~60× coverage per sample on average) (Table 3). Local realignment eliminated 1759 novel variants from the initial call set, representing more than 7% of all novel calls, with a Ti/Tv ratio of 0.77, which indicated that about 90% of these novel calls were false-positives (Figure 2A). In contrast, base quality recalibration eliminated only 446 novel variants with a Ti/Tv ratio of 0.56 but added 306 novel variants with a Ti/Tv ratio of 0.86 from the initial call set (Figure 2B). Marking duplication removed 244 novel variants with a Ti/Tv ratio of 0.97 but it added 107 novel variants with a Ti/Tv ratio of 0.78 from the initial call set (Figure 2C). These results suggested that local realignment was efficient in reducing the false-positive rate, while the effect of recalibration and marking duplications was limited for deep-sequencing data.

**Table 3:** Effects of duplicate marking, realignment & recalibration on SNP calling accuracy

Call set	Site discovery					
	
	No. SNPs				Ti/Tv ratio	
	
	All	Known	Novel	dbSNP%	Known	Novel
**Deep coverage with QUAL > 50**						

initial	96472	71534	24938	74.15%	2.50	1.73
realignment	94595	71374	23221	**75.45%**	2.50	**1.84**
recalibration	96316	71518	24798	74.25%	2.50	1.75
mark duplicate	96303	71502	24801	74.24%	2.50	1.73

**Shallow coverage with QUAL > 20**						

initial	780490	607178	173312	77.79%	2.13	1.39
realignment	776560	606806	169754	78.14%	2.13	1.41
recalibration	783387	609601	173786	77.81%	2.13	1.40
mark duplicate	738198	583829	154369	**79.09%**	2.13	**1.53**

SNPs were called for 5 samples together by GATK using bases with base quality≥20 and reads with mapping quality ≥20. Only sites with QUAL > 50 for deep-coverage or QUAL > 20 for shallow coverage were considered as potentially variable sites.

For low-coverage sequencing (outside regions with > 200 bp distance, ~4× coverage per sample on average), however, the ability of these three steps to eliminate false-positive variants changed. Marking duplication obtained the highest performance with 79.09% dbSNP rate and a novel Ti/Tv ratio of 1.53 (Table 3). Marking duplication removed 19472 novel variants from the initial call set, representing more than 10% of all novel calls, with a Ti/Tv ratio of 0.67 (Figure 2F). In contrast, local realignment only eliminated 4139 novel variants with a Ti/Tv ratio of 0.77 (Figure 2D) and recalibration only removed 3526 novel variants with a Ti/Tv ratio of 0.93 (Figure 2E). These results suggested that marking duplication was more efficient in reducing false-positive rates than other two optional steps for low-coverage sequencing data.

A comprehensive comparison using variable quality thresholds also suggested that realignment was more efficient in removing false positives than base call recalibration and marking duplication for high-coverage data, whereas marking duplication was more efficient than the other two for low-coverage data (Additional file 2).

The effect of orders of the optional steps on SNP calling was also evaluated. We obtained the same accuracy of SNP and genotype calling using different order arrangements, suggesting that the order of steps had no effect on the calling performance (Additional file 3).

### Comparing the performance of GATK, SAMtools and GlfMultiples

**Figure 3:**
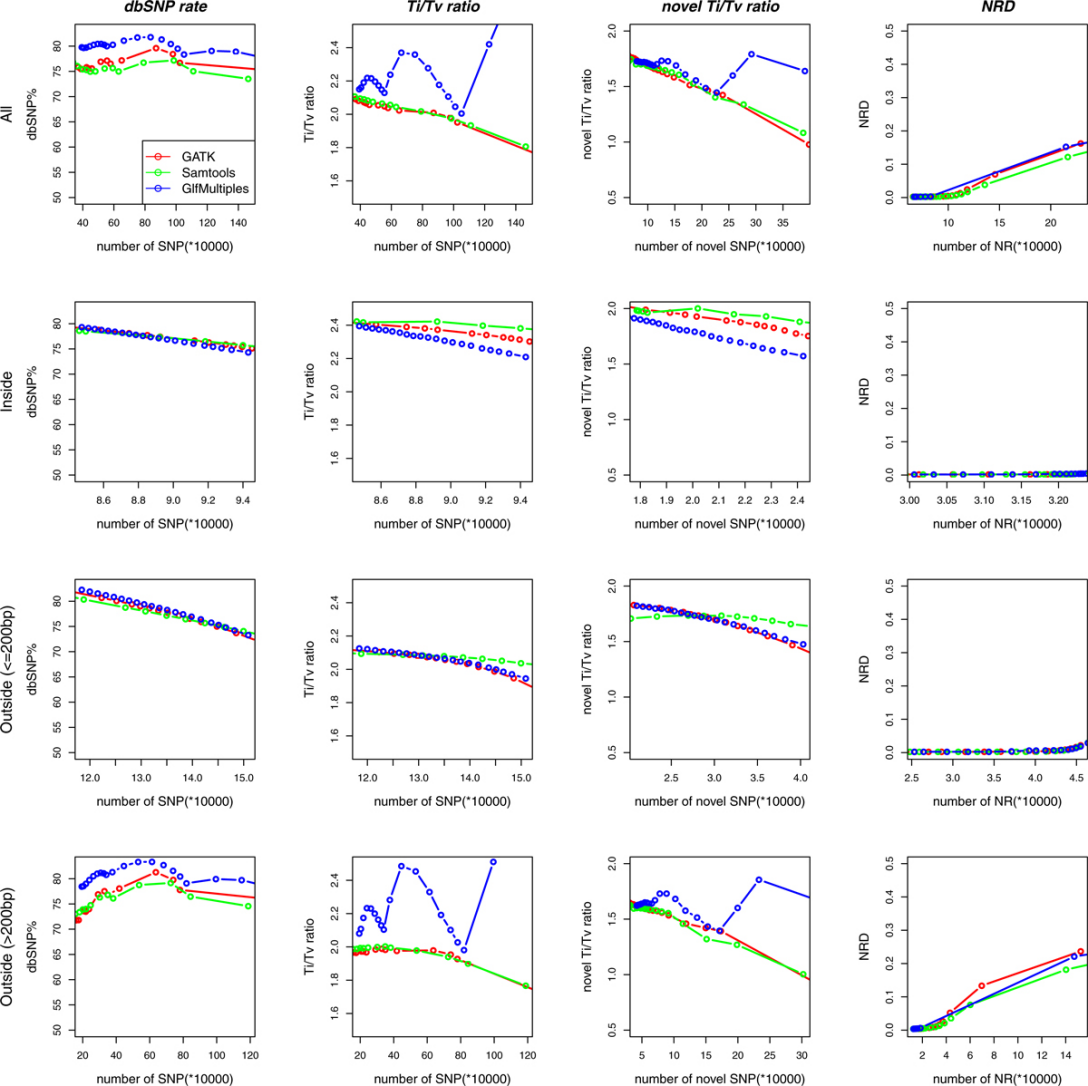
**Comparison of the calling performance of SAMtools, GATK and GlfMultiples in terms of dbSNP rate, Ti/Tv ratio, novel Ti/Tv ratio and NRD (non-reference discrepancy) from all regions, inside target regions, outside target regions with ≤ 200 bp distance and outside target regions > 200 bp distances from Illumina whole-exome sequencing data**.

SAMtools and GATK obtained higher known and novel Ti/Tv ratios than GlfMultiples for deep-sequencing data (inside target regions), while they produced a lower dbSNP rate and known and novel Ti/Tv ratios than GlfMultiples for low-sequencing data (outside regions > 200 bp) when the same number of SNPs were identified (Figure 3). For those data with medium-coverage, these three multi-sample calling tools produced similar dbSNP rate, known and novel Ti/Tv ratios (outside regions ≤ 200 bp). All of these three tools produced a similar genotype concordance with SNP chip data for all regions (Figure 3). These results suggested that SAMtools and GATK had better performance than GlfMultiples for high-coverage data, while GlfMultiples were superior to SAMtools and GATK for low-coverage data.

## Discussion

Intriguingly, we found that the read preprocessing steps before mapping were not necessary. Trimming off low-quality tails from reads even worsen the power of variant calling, although it helps align more reads with high error rate in the tail. A possible explanation is that although the quality of tails is not good enough, they are still helpful for reads mapping. Thus trimming off low-quality tails would lead to more alignment artifacts than using raw reads and, in turn, cause false-positive variants discovery. It should be noted that trimming reads is somehow a question of trial and error and a balance between the number of mapped reads and mapping accuracy. If the decrease of the quality of the 3' end is acceptable and the loss of coverage is affordable, trimming is not necessary. In contrast, if there is a dramatic quality decrease at the tail and poor quality was observed at very earlier sequencing cycle, trimming might be helpful by increasing the number of mapped reads greatly but without reducing the mapping accuracy much.

For the steps after read mapping, including marking duplication, realignment and recalibration, the relative contribution of each step to the accuracy of variant calling depends on the sequencing depth. When the sequencing depth is high, read mapping can benefit from finding consistent alignment among all reads and thus reduce the number of false-positives effectively. When the sequencing depth is low, however, the lack of sufficient reads mapping to the locus limits the power of local multiple sequence alignment and thus it cannot improve the quality of variant calls much. In such circumstances, marking duplication plays a more important role in reducing false positives than realignment and recalibration. Moreover, the performances of three popular multi-sample calling tools, SAMtools, GATK and GlfMultiples, also depend on the sequencing depth. They use the same genotype likelihood model, but GlfMultiples not only takes into account the maximized likelihood but also an overall prior for each type of polymorphism. For example, they favor sites with transition polymorphisms over those with transversion [14]. Thus, incorporating such additional information helps reduce the uncertainty associated with shallow-sequencing data. However, the additional information will disturb the identification of variants when enough evidence is already involved with deep-sequencing data.

The steps posterior to variant calling, including linkage-based genotype refining and SNP filtering or variant quality score recalibration, also contribute a lot to the accurate SNP and genotype calling. The use of LD (linkage-disequilibrium) patterns can substantially improve genotype calling when multiple samples have been sequenced [16]. Because not all information regarding errors can be fully incorporated into the statistical framework, the proper SNP filtering strategies are recommended to reduce the error rates [24]. Besides, the consensus of multiple call sets from different methods provide higher quality than any of individual call sets [14]. Even with the best pipelines, however, we are still far from obtaining a complete and accurate picture of SNPs and genotypes in the human genome. The most challenging task is to distinguish rare variants from sequencing errors. SNP and genotype calling for rare variants, which would not be represented in any reference panel, may not improve much by the use of LD information. To identify rare variants, a direct and more powerful approach is to sequence a large number of individuals [23,32]. In addition to using the proper sequencing strategies, developing more accurate SNP detection methods is needed. More research is also needed in other areas, including longer read depths, improved protocols for generate paired ends, advances in sequencing technology with lower base calling error rates, and more powerful alignment methods.

## Conclusions

Here, we evaluated the effect of a number of computational steps on the accuracy of SNP and genotype calling from Illumina sequencing data with different coverage. To our knowledge, no other study has made a systematic assessment of whether each step is valuable and how it affects the quality of variant detection. Our findings can serves as the general guideline for choosing SNP calling strategies.

## Competing interests

The authors declare that they have no competing interests.

## Authors' contributions

YS led the project and oversaw the analysis. QL and YG designed and performed the research. JL participated in the data analysis. JRL guided the experiments and provided the NGS data. BZ guided the analysis and revised the manuscript. QL wrote the manuscript. All authors have read and approved of the final manuscript.

## Supplementary Material

Additional file 1 **Comparison of effect of different preprocessing steps**. A detailed comparison of calling results with different preprocessing steps in terms of dbSNP rate, Ti/Tv ratio, novel Ti/Tv ratio and NRD for all regions, inside target regions, outside ≤ 200 bp regions, and outside > 200 bp regions from Illumina whole-exome sequencing data. Raw (blue), filterY (green), trim (black) and filterY&trim (red). Click here for file

Additional file 2 **Comparison of effect of marking duplication, realignment and recalibration**. A detailed comparison of results using different steps, marking duplication, realignment and recalibration, in terms of dbSNP rate, Ti/Tv ratio, novel Ti/Tv ratio and NRD for all regions, inside target regions, outside ≤ 200 bp regions, and outside > 200 bp regions from Illumina whole-exome sequencing data. Initial alignment (black), marking duplication (yellow), realignment (violet), recalibration (blue), marking duplication followed by realignment (red), marking duplication followed by realignment and recalibration (brown). Click here for file

Additional file 3 **Comparison of effect of different arrangements of marking duplication, realignment and recalibration**. A detailed comparison of results by arranging three steps, marking duplication, realignment and recalibration, in different orders in terms of dbSNP rate, Ti/Tv ratio, novel Ti/Tv ratio and NRD for all regions, inside target regions, outside ≤ 200 bp regions, and outside > 200 bp regions from Illumina whole-exome sequencing data. Marking duplication followed by realignment and recalibration (red), marking duplication followed by recalibration and realignment (red), realignment followed by recalibration and marking duplication (gray). Click here for file
