# Supplementary figures and images for: Steps to ensure accuracy in genotype and SNP calling from Illumina sequencing data
Source: BMC Genomics. 2012 Dec 17;13(Suppl 8):S8. doi: 10.1186/1471-2164-13-S8-S8 (PMC3535703; doi:10.1186/1471-2164-13-S8-S8)

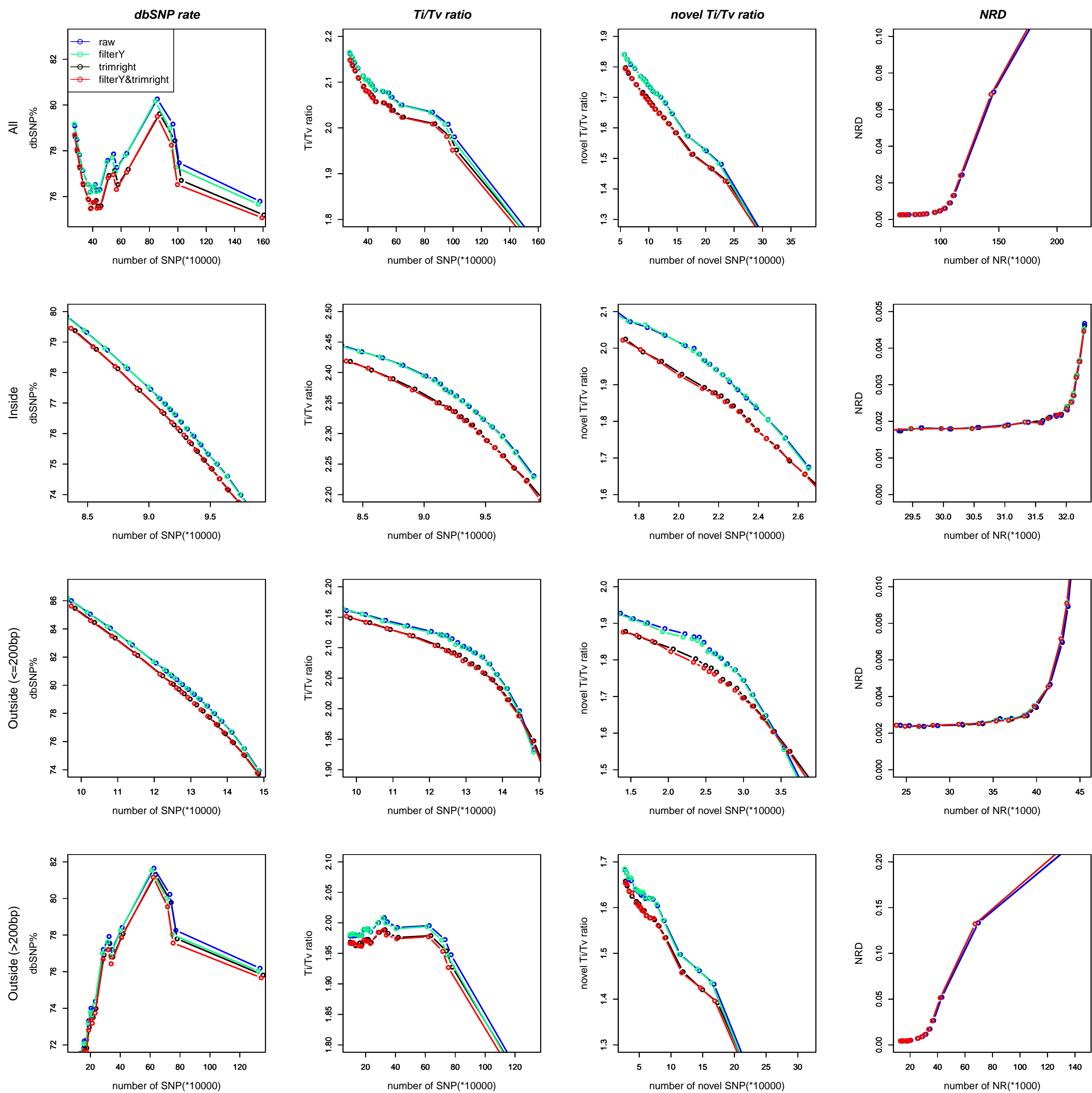

Supplement: Additional file 1 — Comparison of effect of different preprocessing steps. A detailed comparison of calling results with different preprocessing steps in terms of dbSNP rate, Ti/Tv ratio, novel Ti/Tv ratio and NRD for all regions, inside target regions, outside ≤ 200 bp regions, and outside > 200 bp regions from Illumina whole-exome sequencing data. Raw (blue), filterY (green), trim (black) and filterY&trim (red). [file 1471-2164-13-S8-S8-S1.pdf]

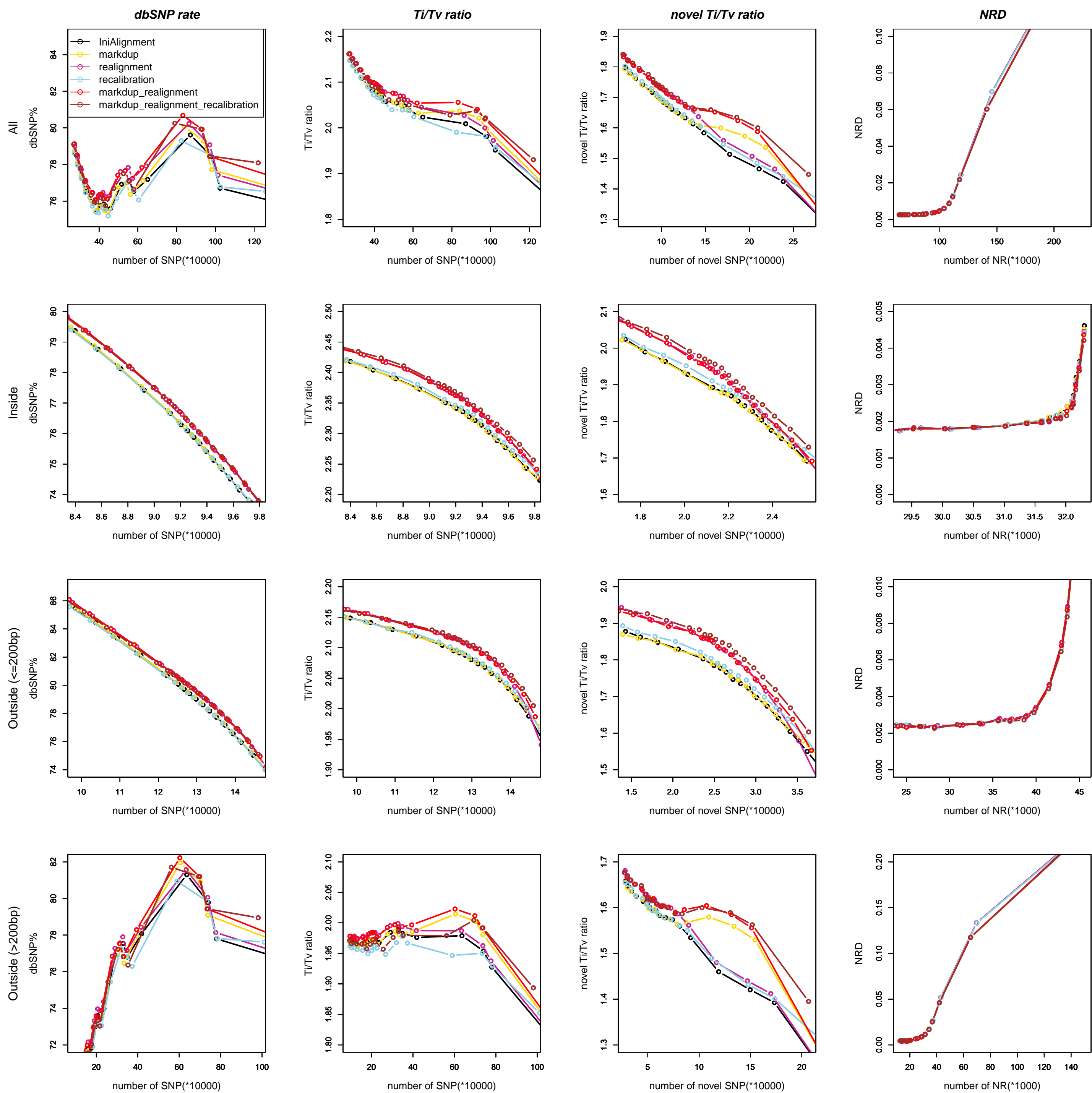

Supplement: Additional file 2 — Comparison of effect of marking duplication, realignment and recalibration. A detailed comparison of results using different steps, marking duplication, realignment and recalibration, in terms of dbSNP rate, Ti/Tv ratio, novel Ti/Tv ratio and NRD for all regions, inside target regions, outside ≤ 200 bp regions, and outside > 200 bp regions from Illumina whole-exome sequencing data. Initial alignment (black), marking duplication (yellow), realignment (violet), recalibration (blue), marking duplication followed by realignment (red), marking duplication followed by realignment and recalibration (brown). [file 1471-2164-13-S8-S8-S2.pdf]

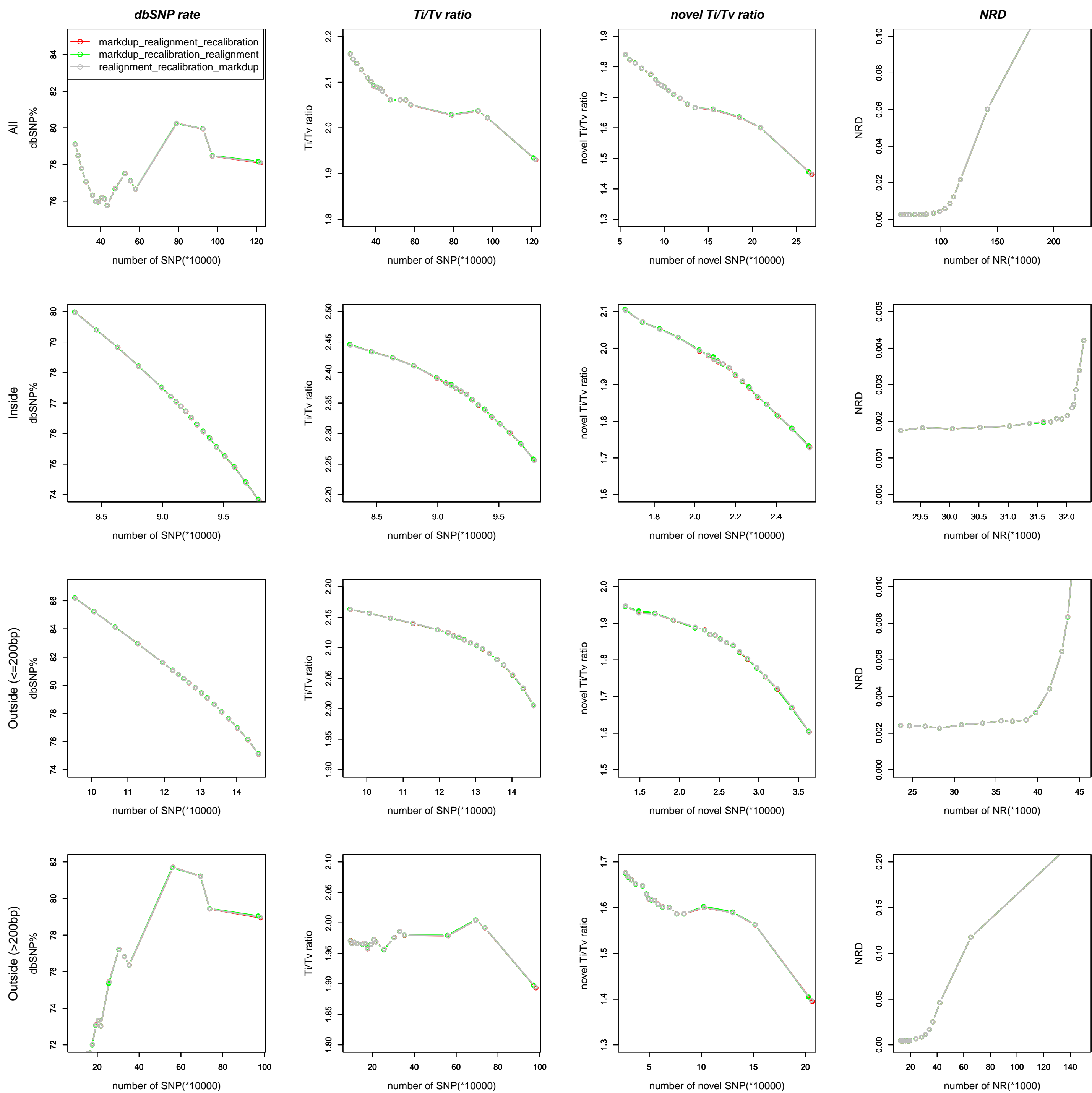

Supplement: Additional file 3 — Comparison of effect of different arrangements of marking duplication, realignment and recalibration. A detailed comparison of results by arranging three steps, marking duplication, realignment and recalibration, in different orders in terms of dbSNP rate, Ti/Tv ratio, novel Ti/Tv ratio and NRD for all regions, inside target regions, outside ≤ 200 bp regions, and outside > 200 bp regions from Illumina whole-exome sequencing data. Marking duplication followed by realignment and recalibration (red), marking duplication followed by recalibration and realignment (red), realignment followed by recalibration and marking duplication (gray). [file 1471-2164-13-S8-S8-S3.pdf]
